# Adjuvant Chemotherapy Is Associated With Prolonged Survival Time in Small‐Breed Dogs Undergoing Amputation for Appendicular Osteosarcoma

**DOI:** 10.1111/vco.13041

**Published:** 2025-01-11

**Authors:** Stefano Zanardi, Silvia Sabattini, Federica Rossi, Matteo Rossanese, Paolo Buracco, Vincenzo Montinaro, Marina Aralla, Alfredo Dentini, Elisa Pizzi, Enrico Volpe, Giovanni Tremolada, Laura Marconato

**Affiliations:** ^1^ Department of Veterinary Medical Sciences University of Bologna, Ozzano dell'Emilia Bologna Italy; ^2^ Anicura Clinica Veterinaria dell'Orologio Bologna Italy; ^3^ Department of Clinical Science and Services The Royal Veterinary College Hatfield UK; ^4^ Department of Veterinary Science University of Turin Turin Italy; ^5^ Anicura Clinica Veterinaria Malpensa Varese Italy; ^6^ Pronto Soccorso Veterinario Laudense Lodi Italy; ^7^ Anicura Clinica Veterinaria Tyrus Terni Italy; ^8^ Clinica Veterinaria Concordia Venezia Italy; ^9^ Flint Animal Cancer Center Colorado State University Fort Collins Colorado USA

**Keywords:** amputation, canine, chemotherapy, osteosarcoma, prognosis, small breed

## Abstract

Adjuvant chemotherapy is a well‐established treatment for large‐breed dogs with appendicular osteosarcoma; however, it is unclear if it improves outcomes in small‐breed dogs due to limited focused studies. This retrospective study aimed to investigate the oncologic outcomes of dogs weighting less than 15 kg with appendicular osteosarcoma that underwent curative resection with or without postoperative adjuvant chemotherapy. Endpoints were time to distant progression (TTDP) and overall survival (OS). Medical records from multiple institutions were reviewed, and 43 dogs were included in the analysis: 17 underwent surgery alone and 26 also received adjuvant chemotherapy. The median TTDP for all dogs was 265 days, with no significant difference between treatment groups. The median OS for all dogs was 270 days, and it was significantly different between amputated dogs (150 days) and those also receiving adjuvant chemotherapy (353 days, *p* = 0.002). In our cohort, osteosarcoma in small breeds behaved as aggressive as in large breeds. Adjuvant chemotherapy may prolong survival. Future randomised studies are needed to provide definitive evidence on the necessity of adjuvant chemotherapy to address metastatic spread in small‐breed dogs with appendicular osteosarcoma.

## 1 Introduction

Osteosarcoma is the most common bone tumour found in dogs, primarily affecting the appendicular skeleton [1]. Although appendicular osteosarcoma can occur in any breed [2], it notably targets large and giant breeds more often than small breeds, with the latter constituting less than 5% of cases [3].

Curative‐intent treatment for osteosarcoma typically involves either amputation of the affected limb or *en bloc* excision of the lesion (limb‐sparing surgery), followed by adjuvant chemotherapy [2, 4]. The latter typically targets micrometastatic disease, which is present in 90% of dogs at diagnosis [1, 2, 4].

According to a previous study, small‐breed dogs undergoing amputation alone showed an extended median survival time compared to large‐breed dogs with similar conditions [3]. Notably, there was no significant survival benefit for dogs undergoing curative‐intent treatment (median, 415 days) compared to those undergoing amputation alone (median, 257 days) [3]. Thus, a critical question regarding small‐breed dogs is whether their risk of developing metastases is equivalent to that of larger individuals, which would result in the recommendation for adjuvant chemotherapy.

The aim of this multicentre retrospective study was to evaluate the benefit of adjuvant chemotherapy in small‐breed dogs with osteosarcoma in terms of time to distant progression (TTDP) and overall survival (OS). We hypothesised that OS and response to chemotherapy in small‐breed dogs with appendicular osteosarcoma would be comparable to those observed in large‐breed dogs.

## 2 Materials and Methods

The current study was designed by the Italian Society of Veterinary Oncology (SIONCOV). The medical databases of 11 veterinary centres, including veterinary teaching hospitals and private practises, were retrospectively reviewed to identify dogs weighing < 15 kg with histologically confirmed appendicular osteosarcoma treated with surgery alone or followed by adjuvant chemotherapy between 2014 and 2024. Medical records and reported data were reviewed for inclusion by the authors.

In compliance with local legislation, ethical approval was not required for this study. All owners provided written informed consent for the use of their dogs' medical records.

Dogs were included if they underwent surgery and had osteosarcoma confirmed by histopathologic examination. Medical records needed to be complete regarding initial staging findings, as detailed below, and follow‐up information had to be accessible for at least 6 months following diagnosis for dogs not deceased from tumour‐related causes.

Dogs were not included if osteosarcoma involved the metacarpal/metatarsal or digital bones. Additional exclusion criteria were axial and extraskeletal locations, incomplete staging information or previous antitumoural treatments.

Data obtained from the medical records included signalment (breed, age, sex, body weight), clinical signs at diagnosis, duration of clinical signs, date of diagnosis, osteosarcoma location, imaging modalities utilised at the time of staging (thoracic radiographs and abdominal ultrasound or total body computed tomography [TBCT]) and their results, haematology and serum biochemistry results at admission, surgical treatment, occurrence and location of distant metastasis (if any) and/or local recurrence, date of death, cause of death if known, and date of last follow‐up.

Tumour‐specific variables, when available from the histopathology reports, included subtype (osteoblastic, telangiectatic, chondroblastic, fibroblastic), grade according to Loukopoulos and nodal status [5].

If administered, types of adjuvant treatments (drugs and dosages), time interval between surgery and initiation of chemotherapy, and treatment‐related toxicity were recorded [6].

At the documentation of progression, any rescue treatments attempted were also recorded.

The recheck schedule, imaging modality for restaging purposes, and monitoring diagnostics were determined at the discretion of the treating clinician. It was recommended to monitor pulmonary metastasis every 2–3 months, unless clinical signs suspicious for metastasis were present, in which case imaging was carried out sooner. Follow‐up abdominal ultrasound occurred according to each clinician's and owner's preference.

Patient status was recorded as alive at data analysis closure, dead, or lost to follow‐up. Cause and date of death were recorded, if known, from the medical record or after email correspondence or phone calls with the referring veterinarian.

### 2.1 Statistical Analysis

Descriptive statistics were used in the analysis of dogs and tumour characteristics. When appropriate, data were tested for normality using the D'Agostino and Pearson omnibus normality test. Values were expressed as mean ± SD in case of normal distribution, or as median with a range in case of non‐normal distribution.

Time to distant progression (TTDP) was defined as the time from surgery to the date of presumed or confirmed new metastatic lesions or progression of existing ones. OS was defined as the time from surgery to the date of death from any cause. Dogs not experiencing distant progression or death at the end of the study period were censored from the respective analysis.

Survival estimates were presented as medians with the corresponding 95% CI.

The influence of potential prognostic variables on TTDP and OS was investigated with Cox's regression analysis. The following variables were investigated for prognostic significance: sex, age (median used as cut‐off), body weight (median used as cut‐off), clinical signs duration (median used as cut‐off), osteosarcoma location (site and proximal versus distal involvement), type of imaging (thoracic radiographs and abdominal ultrasound versus TBCT), distant metastasis at diagnosis (yes or no), serum alkaline phosphatase (ALP) level (normal versus increased), monocyte count (≤ 0.4 × 10^3^ cells/μL vs. > 0.4 × 10^3^ cells/μL) [7], lymphocyte count (≤ 1.0 × 10^3^ cells/μL vs. > 1.0 × 10^3^ cells/μL) [7], histologic subtype, histologic grade, histologic presence of lymph node metastasis (yes or no), and medical treatment (yes or no). The prognostic relevance of adjuvant chemotherapy was further assessed using the log‐rank test.

Statistical analysis was performed with SPSS Statistics v.19 (IBM, Somers, New York). Significance was set at *p* ≤ 0.05.

## 3 Cell Line Validation Statement

No cell lines were used in the current study.

## 4 Results

### 4.1 Dogs and Tumour Characteristics

The database search identified 49 dogs diagnosed with presumed appendicular osteosarcoma, all of which were evaluated for eligibility. Dogs were excluded from the analysis due to incomplete clinical records (*n* = 3), or because they did not undergo surgery (*n* = 3).

In total, 43 dogs with confirmed appendicular osteosarcoma were included in the analysis. The main demographic information is summarised in Table 1.

**TABLE 1:** Demographic information recorded in 43 dogs weighting less than 15 kg with appendicular osteosarcoma.

Breed	
Mixed breed	21 (48.8%)
Pure‐breed	22 (51.2%)
Jack Russell terrier	5 (11.6%)
French bouledogue	3 (6.9%)
Miniature schnauzer	3 (6.9%)
Fox terrier	2 (4.7%)
Border collie	1 each (2.3%)
Bichon frisee	
Breton	
Cairn terrier	
Cocker spaniel	
Lagotto romagnolo	
Pomeranian dog	
Whippet	
Dachshund	
Median age (range)	11 (3–14) years
Sex	
Male (neutered)	26 (14)
Female (spayed)	17 (12)
Median weight (range)	11 (5–15) kg

All dogs were lame at the time of presentation with a median time interval of 30 days (range, 1–150) from the onset of clinical signs to diagnosis. Twenty‐six dogs (60.5%) had a clinical signs duration of 30 days or less (range, 1–30), while 17 dogs (39.5%) had a clinical signs duration greater than 30 days (range, 34–150).

By descending order, osteosarcoma involved the tibia (*n* = 18, 41.9%; 9 distal, 8 proximal, 1 diaphyseal), followed by femur (*n* = 12, 27.9%; 11 distal and 1 diaphyseal), humerus (*n* = 5, 11.6%; 3 distal and 2 proximal), radius (*n* = 5, 11.6%; all distal), scapula (*n* = 2, 4.7%) and tarsus (*n* = 1; 2.3%). Two (4.7%) dogs experienced a pathologic fracture, and in one (2.3%) dog osteosarcoma developed at a site of a previously applied orthopaedic implant.

Imaging techniques adopted for staging included TBCT in 32 (74.4%) dogs, while the remaining 11 (25.6%) underwent 3‐view thoracic radiographs and abdominal ultrasound. At the time of diagnosis, five (11.6%) dogs had detectable lung metastasis.

Serum ALP concentration value was available for 35 (81.4%) dogs. Among them, 11 (31.4%) had an increased level. The monocyte count was available for 33 (76.7%) dogs, of which five (15.2%) had > 0.4 × 10^3^ monocytes/μL. The lymphocyte count was available for 36 (83.7%) dogs, of which 6 (16.7%) had > 1.0 × 10^3^ lymphocytes/μL.

### 4.2 Surgery and Histopathologic Findings

Overall, 42 (97.7%) dogs underwent limb amputation and 1 (2.3%) had limb‐sparing surgery.

Limb amputation consisted of coxofemoral disarticulation (*n* = 29), forelimb amputation including the scapula (*n* = 10), scapulohumeral disarticulation (*n* = 2) and amputation at the level of the distal third of the femur (*n* = 1).

Regional lymphadenectomy was concurrently performed in 41 (95.3%) dogs, involving the removal of the axillary and cervical superficial lymph nodes for the thoracic limb and inguinal superficial lymph nodes for the pelvic limb.

Through histopathologic evaluation osteosarcomas were subtyped as osteoblastic (*n* = 34), fibroblastic (*n* = 5), chondroblastic (*n* = 3) and poorly differentiated (*n* = 1). A histologic grade was assigned to 37 (86.0%) cases, with 8 (21.6%) classified as grade 1, 22 (59.5%) as grade 2, and 7 (18.9%) as grade 3.

Two out of 41 (4.9%) dogs had histologically confirmed nodal metastases.

### 4.3 Adjuvant Treatment

Overall, 26 (60.5%) dogs received adjuvant chemotherapy after a median time interval from amputation of 15 days (range, 1–31).

Eighteen (69.2%) dogs received carboplatin alone (median, 4 doses; range, 1–7), five (19.3%) received doxorubicin alone (median, 4 doses; range, 2–4) and three (11.5%) received alternating doses of carboplatin (median, 2 doses; range, 2–3) and doxorubicin (median, 2 doses; range, 1–3).

Carboplatin was administered at a median dose of 280 mg/m^2^ (range, 240–300) and doxorubicin was administered at 1 mg/kg to all dogs.

Maintenance treatment at the completion of dose‐intense chemotherapy was administered to eight dogs, consisting of immunotherapy (*n* = 7) and metronomic cyclophosphamide (*n* = 1).

Eleven (25.6%) dogs experienced chemotherapy‐related adverse events. Overall, there were seven episodes of adverse events involving the bone marrow (*n* = 4 grade 1, *n* = 1 grade 2 and *n* = 2 grade 4), four episodes of toxic effects involving the gastrointestinal tract (*n* = 2 grade 1 and *n* = 2 grade 2) and one episode of grade 2 toxic effects involving renal function.

At the documentation of metastatic development, two dogs received rescue treatment, consisting of two doses of doxorubicin and three doses of doxorubicin followed by toceranib and metronomic cyclophosphamide, respectively. One dog survived an additional 150 days, while the other lived only 30 days.

Seventeen (39.5%) dogs (of which four with distant metastasis) received no adjuvant chemotherapy.

Demographic and clinical information stratified by treatment are presented in Table S1.

### 4.4 Outcome Data and Prognostic Factors

During follow‐up, 26 (58.1%) dogs developed new metastatic disease to lungs (*n* = 19), bone (*n* = 5), muscular/subcutaneous (*n* = 3) and spleen and liver (*n* = 2). All dogs with metastatic disease at admission experienced progression of their metastatic lesion. Metastatic lesions, whether newly developed or pre‐existing and progressing, were suspected based on imaging in all dogs, with only five of them undergoing cytologic confirmation. Specifically, these five dogs developed visceral, skeletal, and/or cutaneous metastases, which were sampled.

**FIGURE 1:**
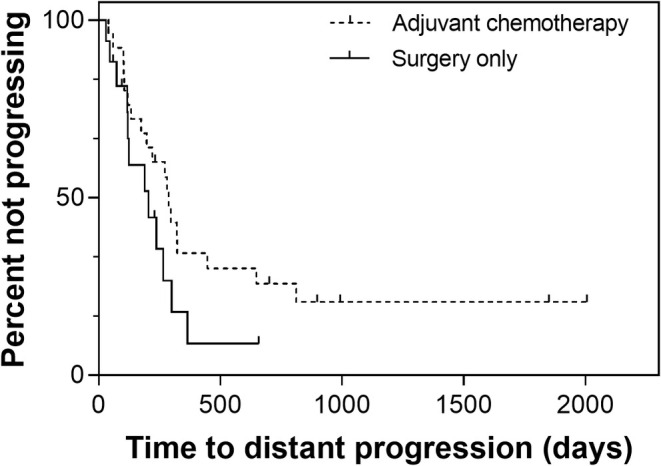
Time to distant metastasis (TTDP) for 43 small‐breed dogs with osteosarcoma treated with surgery only (solid line) or surgery with adjuvant chemotherapy (dashed line). Tick marks represent censored patients. TTDP is not significantly longer for dogs receiving adjuvant chemotherapy (HR: 1.8; 95% CI: 0.8–3.7; *p* = 0.134).

The median TTDP for all dogs was 265 days (95% CI, 179–351). Though not statistically significant (*p* = 0.129), the median TTDP was 288 days (95% CI, range, 248–328) for dogs receiving adjuvant chemotherapy, and 205 days (95% CI, 64–346) for dogs undergoing limb amputation only (Figure 1; Table 2).

**TABLE 2:** Outcome information recorded in 43 small‐breed dogs with appendicular osteosarcoma treated with curative intent surgery with or without adjuvant chemotherapy.

Variable	All dogs (*n* = 43)	Adjuvant chemotherapy (*n* = 26)	Surgery only (*n* = 17)	*P*
Median TTDP (95% CI) Excluding dogs with distant metastasis at diagnosis	265 (179–351) d 281 (182–380) d	288 (248–328) d 288 (249–327) d	205 (64–346) d 205 (84–325) d	0.129 0.370
Median OS (95% CI) Excluding dogs with distant metastasis at diagnosis	270 (191–349) d 297 (220–374) d	353 (131–575) d 353 (64–641) d	150 (9–291) d 150 (29–271) d	0.002^a^ 0.012^a^
1‐year survival rate	35.7%	50%	19.2%	0.014^a^
2‐year survival rate	11.4%	12.5%	0%	0.138

Abbreviations: CI: confidence interval; d: days; OS: overall survival; TTDP: time to distant progression.

^a^ Significant.

Upon removal of dogs with distant metastases at diagnosis, the TTDP was not significantly different between dogs receiving (median, 288 days; 95% CI, 249–327) and not receiving (median, 205 days; 95% CI, 84–325) adjuvant therapy (*p* = 0.370; Table 2).

At data analysis closure, 38 (88.4%) dogs had died and 5 (11.6%) were still alive after a median follow‐up time of 701 days (range, 229–992). Death was tumour‐related in 33 (86.8%) dogs and tumour‐unrelated in 5 (13.2%) dogs. Causes of tumour‐related death included the development or progression of metastatic disease (*n* = 31), chemotherapy‐related septicaemia (*n* = 1) and local recurrence of a scapular osteosarcoma (*n* = 1). Causes of unrelated death included splenic hemangiosarcoma, granulosa cell tumour, heatstroke, car accident and metabolic comorbidities (concurrent hyperadrenocorticism and diabetes).

The median OS for all dogs was 270 days (95% CI, 191–349). The median OS significantly differed among treatment groups: 353 days (95% CI, 131–575) for dogs also receiving adjuvant therapy and 150 days (95% CI, range, 9–291) for dogs undergoing amputation only (*p* = 0.002; Figure 1). Upon removal of dogs with distant metastases at diagnosis, the outcome was still significantly different between dogs receiving (median OS, 353 days; 95% CI, 64–641) and not receiving (median OS, 150 days; 95% CI, 29–271) adjuvant therapy (*p* = 0.012; Table 2). Dogs receiving immunotherapy in addition to chemotherapy did not experience significantly longer TTDP (median not reached vs. 288 days; *p* = 0.129) or OS (630 vs. 306 days; *p* = 0.139) compared to those receiving chemotherapy alone.

The overall 1‐ and 2‐year survival rates were 35.7% and 11.4%, respectively; 50% and 19.2% for dogs receiving chemotherapy and 12.5% and 0% for dogs receiving amputation only (Table 2).

**FIGURE 2:**
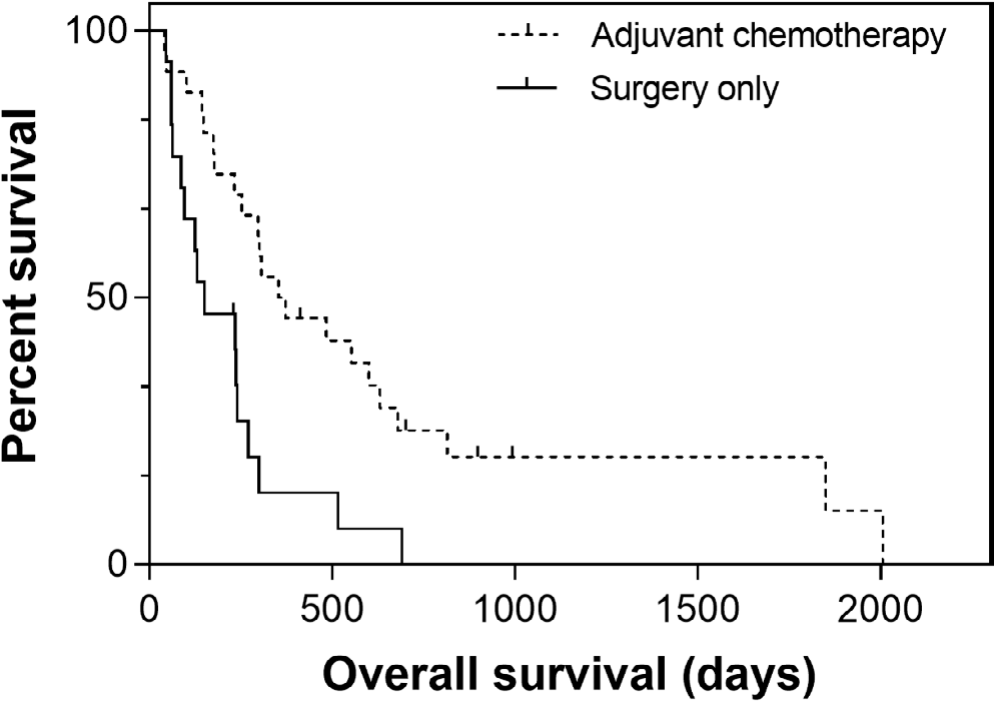
Overall survival (OS) for 43 small‐breed dogs with osteosarcoma treated with surgery only (solid line) or surgery with adjuvant chemotherapy (dashed line). Tick marks represent censored patients. OS is significantly longer for dogs receiving adjuvant chemotherapy (HR: 2.8; 95% CI: 1.4–5.7; *p* = 0.003).

The only variable significantly associated with an increased risk of distant progression was the presence of distant metastatic disease at diagnosis (HR: 3.3; 95% CI, 1.2–9.1; *p* = 0.021), whereas lack of adjuvant therapy (HR: 2.8; 95% CI, 1.4–5.7; *p* = 0.003) was significantly associated with an increased risk of death (Figure 2).

## 5 Discussion

In this retrospective study including 43 dogs weighing less than 15 kg with appendicular osteosarcoma undergoing surgery, adjuvant chemotherapy notably increased survival time. The role of chemotherapy in improving outcome has been previously questioned, as no survival benefit was documented in small‐breed dogs undergoing curative‐intent treatment compared to those undergoing amputation alone [3], prompting the need for this study.

Based on the current findings, adjuvant chemotherapy significantly prolonged survival time (353 vs. 150 days) and showed similar toxicity rates and efficacy to those observed in large‐breed dogs [8, 9].

The cause of death was attributable to metastatic disease in 94% of dogs that succumbed to tumour‐related causes. This high percentage aligns with the outcomes observed in large‐breed dogs, where metastatic progression is also a predominant factor in mortality [10].

There is no substantial evidence to suggest that osteosarcoma would behave differently in small breeds compared to large breeds for several reasons. First, the fundamental biologic processes driving tumour growth, metastasis, and resistance to treatment are consistent, regardless of the dog's size [10, 11]. The same holds true for human patients, where the risk of metastasis is influenced by the tumour's biologic characteristics rather than the patient's size or demographics [12, 13]. Second, the histologic features of osteosarcoma are similar in both small and large breeds, contributing to the uniformity in how the disease manifests and progresses [14]. Third, in the current series, 5 dogs had detectable lung metastasis at the time of diagnosis, and an additional 26 dogs developed metastatic lesions during follow‐up. This finding is consistent with previous studies on large breeds, where metastatic disease was documented to develop in 85%–90% of dogs [10, 15]. Last, survival rates and clinical outcomes for osteosarcoma are generally comparable across breeds. If undergoing amputation alone, large‐breed dogs had a median survival time of 119–175 days [16, 17, 18, 19], which is comparable to the data obtained in this study. By contrast, large breeds undergoing adjuvant chemotherapy had a median survival of approximately 1 year [20], mirroring our data on small breeds undergoing curative‐intent treatment. All these consistencies underscore the aggressive nature of osteosarcoma, and highlights that the underlying mechanisms of the disease are similar, regardless of the dog's size.

The same deduction applies to other types of tumours, such as canine oral melanoma and mammary tumours, which have a known predisposition for small breeds, or hemangiosarcoma, which is more common in large breeds [21, 22, 23, 24]. However, the primary prognostic factors for all these tumours are related to the tumour's biology rather than to the patient's size [25].

At the documentation of distant metastasis, two dogs received rescue treatment. One dog survived an additional 150 days and the other lived only 30 days. While the one case surviving an additional 150 days offers some perspective, it is difficult to determine whether the additional survival time was due to chemotherapy or other factors, such as the individual biology of the tumours or patient‐specific response. Larger studies are needed to assess the true efficacy of chemotherapy after distant progression.

In large‐breed dogs, several prognostic factors have been documented, including the specific appendicular bone site affected, with the humerus being associated with a worse outcome, and elevated ALP levels at diagnosis [26]. In the current study the tibia, followed by the femur, humerus and radius were more commonly affected, mirroring the preferential appendicular sites seen in large breeds. However, when comparing thoracic to pelvic limbs, only 27.9% of cases in our series involved the forelimb, in contrast to a larger study of 744 dogs, where thoracic limb involvement was reported in 64.2% of cases [27]. Also, the skeletal site was not associated with an increased risk of tumour‐related death. Although this may be due to a type II error, a different biologic behaviour cannot be completely ruled out. Likewise, nearly one third of small‐breed dogs in the current study had elevated ALP levels at diagnosis, but this was not associated with a poorer outcome. Breed‐related variations in ALP levels have been reported [28, 29]; thus, further research is needed to clarify the prognostic role of ALP, as has been documented in large breeds.

In large‐breed dogs, the presence of nodal metastasis is also associated to a poorer prognosis [30]. In the current series, two dogs presented with nodal metastasis at the time of diagnosis, one of which also had pulmonary metastasis. Both dogs underwent lymphadenectomy and experienced disease progression. The dog with both nodal and pulmonary metastasis died after 237 days due to worsening pulmonary lesions. The second dog died after 483 days due to lung and skeletal metastases. Although a previous study has addressed the prognostic impact of nodal metastasis, definitive conclusions cannot be drawn from this cohort due to the small number of dogs with lymph node involvement.

The results obtained here differ from a previous study also focusing on small‐breed dogs, but this paper aims to complement rather than contradict that study [3]. While both studies have similar population sizes and outcomes, notable methodologic differences exist. The Amsellem study primarily relied on thoracic radiographs for staging, whereas 74% of dogs in our cohort underwent TBCT, suggesting the possibility that dogs with undetected lung metastasis could have been included in the prior study, potentially impacting survival outcomes. Additionally, only one dog in the Amsellem study underwent lymphadenectomy with histologic analysis, compared to 95% in our series. Lastly, the prior study also included four dogs with digital, metatarsal, or metacarpal osteosarcoma which may have impacted results given the typically less aggressive biological behaviour of these locations. However, a type‐II error due to the small sample size cannot be excluded.

In this cohort, chemotherapy was associated with improved overall survival but did not delay the onset of distant progression. This suggests that while chemotherapy may not prevent the initial development of metastases, it could slow the growth or reduce their impact on survival once they appear. Additionally, chemotherapy might support immunosurveillance by enhancing antitumor immunity, thus extending survival despite a similar time to metastatic onset [31, 32]. Alternatively, chemotherapy could be selectively effective against sensitive cancer cells, potentially prolonging survival by controlling only susceptible cells without affecting the timing of detectable metastasis. Additionally, finances and euthanasia can significantly impact survival outcomes in veterinary patients. Owners who chose adjuvant chemotherapy may have also been more likely to pursue rescue therapies and palliative care, while those who declined adjuvant treatments may have opted for earlier euthanasia.

Several limitations must be acknowledged, primarily due to the retrospective nature of the study. Despite involving 11 centres over a 10‐year period, the study population was small, confirming that osteosarcoma is relatively rare in small breeds, thus making case accrual challenging. The participating centres had differing record‐keeping practises, which may have led to missing or inaccurate data. The study relied on medical records, which could introduce bias due to incomplete or inconsistent documentation and a lack of standardisation in diagnostic and therapeutic protocols. When chemotherapy was administered, there was variability in the drugs used, number of cycles, and dosages. Although previous reports have shown similar outcomes across different protocols, doses, and treatment intervals [9, 33], it remains to be verified if this holds true for small breeds. Last, four out of the five dogs with metastatic disease at presentation did not receive any adjuvant chemotherapy, likely due to the perception of a poor prognosis. Although the lack of medical treatment may have contributed to the shorter survival observed in this group, the poor outcome persisted even when excluding dogs with distant metastases at diagnosis.

In conclusion, our study found that osteosarcoma in small‐breed dogs exhibits an aggressive behaviour similar to that observed in large breeds. The current findings suggest that adjuvant chemotherapy may prolong survival and continued efforts are needed to improve our understanding and treatment of this condition. While we hope that future randomised studies may provide definitive evidence for optimal management strategies in this specific population of dogs, we acknowledge the significant challenges posed by the rarity of this condition and the inherent difficulties in conducting such studies. Therefore, we believe that progress will more likely depend on multicentre collaborative efforts and large retrospective analyses.

## Ethics Statement

In compliance with local legislation, ethical approval was not required for this study. Dogs were treated according to the current standards. All owners signed a written informed consent.

## Conflicts of Interest

The authors declare no conflicts of interest.

## Supporting information


**Table S1.** Demographic and clinical information recorded in 43 small‐breed dogs with appendicular osteosarcoma stratified by treatment.

## Data Availability

The data that support the findings of this study are available from the corresponding author upon reasonable request.
