# Supplementary material for: Adjuvant Chemotherapy Is Associated With Prolonged Survival Time in Small‐Breed Dogs Undergoing Amputation for Appendicular Osteosarcoma
Source: Vet Comp Oncol. 2025 Jan 11;23(2):161–7. doi: 10.1111/vco.13041 (PMC12082752; doi:10.1111/vco.13041)
Supplement: Supplementary file 1 — Table S1. Demographic and clinical information recorded in 43 small‐breed dogs with appendicular osteosarcoma stratified by treatment. [file VCO-23-161-s001.docx]

**Supplemental Table 1.** Demographic and clinical information recorded in 43 small-breed dogs with appendicular osteosarcoma stratified by treatment.

|  | **Adjuvant chemotherapy**  (n=26) | **Surgery only**  (n=17) |
| --- | --- | --- |
| Median age (range) | 11 (3-14) years | 11 (3-14) years |
| Sex  male (neutered)  female (spayed) | 15 (9)  11 (9) | 11 (5)  6 (3) |
| Median weight (range) | 10.5 (5-15) kg | 12 (7.5-15) kg |
| Median symptom duration (range) | 21 (1-135) days | 32 (14-120) days |
| Increased ALP (%) | 5 (20%) | 6 (60%) |
| Increased monocyte count (%) | 2 (8.7%) | 3 (30%) |
| Increased lymphocyte count (%) | 4 (16.7%) | 2 (16.7%) |
| Distant metastasis at diagnosis (%) | 1 (3.8%) | 4 (23.5%) |
